# Allocation of photosynthesized carbon in an intensively farmed winter wheat–soil system as revealed by ^14^CO_2_ pulse labelling

**DOI:** 10.1038/s41598-018-21547-y

**Published:** 2018-02-16

**Authors:** Zhaoan Sun, Qing Chen, Xiao Han, Roland Bol, Bo Qu, Fanqiao Meng

**Affiliations:** 1 Beijing Key Laboratory of Farmland Soil Pollution Prevention and Remediation, College of Resources and Environmental Sciences, China Agricultural University, Beijing, 100193 China; 2 Institute of Bio- and Geosciences, Agrosphere Institute (IBG-3), Forschungszentrum Jülich GmbH, 52425 Jülich, Germany

## Abstract

Understanding the rhizodeposited carbon (C) dynamics of winter wheat (*Triticum aestivum* L.), is crucial for soil fertility and C sequestration. Pot-grown winter wheat was pulse labelled with ^14^CO_2_ at the key growth stages. ^14^C in the shoots, roots and soil was measured at 5 or 2 days after ^14^C-labelling (DAL 5/2) at each growth stage and at harvest. The ^14^C in the shoots increased from 4% of the net ^14^C recovered (shoots + roots + soil) during tillering to 53% at harvest. Approximately 14–34% of the net ^14^C recovered was incorporated into the soil. Allocation of photosynthesized C was extrapolated from the pot experiment to field condition, assuming a planting density of 1.8 million plants ha^−1^. The estimated C input to the soil was 1.7 t C ha^−1^, and 0.7 t C ha^−1^ of root residues was retained after wheat harvest; both values were higher than those previously reported (0.6 and 0.4 t C ha^−1^, respectively). Our findings highlight that C tracing during the entire crop season is necessary to quantify the temporal allocation of photosynthesized C, especially the contribution to soil carbon in intensified farming system.

## Introduction

Wheat covers approximately 220 million hectares of farmland worldwide^1^ and plays an important role in food supply and soil organic carbon (SOC) regulation^2^. SOC is primarily derived from plants^3^, such as deposition of both crop straw^4^ and root litter^5,6^, as well as rhizodeposits^3^. Rhizodeposits include root exudates and other root-borne organic substances released into the rhizosphere during plant growth as well as sloughed root hairs and decaying root cells^3,7^; and it also affects SOC and nutrient cycling^8–11^. Accurate investigation of rhizodeposits during the entire wheat season is crucial important for the achievement of high crop yield and understanding of its contribution to farmland C sequestration, especially in intensified agricultural region such as northern China^12,13^.

Types and developmental stage of crop growth significantly influences the distribution of photosynthesized C. Mathew *et al*.^14^ found that grasses can store up to 45% of their C stocks in the roots, while cereals show much lower rates with 16% for maize and 23% for wheat. The belowground allocation of photosynthesized C to the soil decreases from approximately 10% at tillering to 5% at grain-filling in wheat and barley (Table 1). This decrease might be attributed to differences in photosynthetic capacity and C requirements for root growth at different growth stages^3,11^. Extrapolating the values from short periods to the whole lifetime of wheat plants (approximately 240 days after emergence in northern China) might overestimate the amount of C allocated to the belowground pool, as young plants exhibit faster root growth and greater sink strength of C allocated to the roots and soil than do older plants^15,16^.

**Table 1:** Partitioning (%) of pulse-labelled carbon (C) (mean ± SE) at different growth stages of wheat and barley.

	Plant age (days)	Growth stage	Shoots	Roots	Soil	Soil/(soil + roots)	References
Wheat	42	Tillering	70.9	17.6	11.5	39.6	Keith *et al*.^25^
Wheat	50		79.3	20.2	0.5	2.3	Gregory and Atwell^24^
Wheat	43		54.2	26.8	19.0	41.5	Palta and Gregory^43^
Wheat	49		80.8		19.2		Martin and Kemp^44^
Wheat	166		54.2	23.7	22.1	48.2	Swinnen *et al*.^29^
Wheat	37		53.7	43.0	3.2	6.9	Lodhi *et al*.^45^
Barley	49		60.6	37.0	2.4	6.1	Gregory and Atwell^24^
Barley	34		78.0	13.8	8.2	37.3	Jensen^46^
Barley	47		56.5	33.7	9.8	22.5	Swinnen *et al*.^29^
		Average^a^	63.4 ± 3.6	27.0 ± 3.4	9.6 ± 2.6	25.5 ± 6.1	
**Wheat**	**23**	**This study**	**36.4**	**10.8**	**52.9**	**83.0**	
Wheat	77	Elongation	81.2	11.8	7.0	37.1	Keith *et al*.^25^
Wheat	71		75.2	23.4	1.5	5.9	Gregory and Atwell^24^
Wheat	51		53.7	27.4	18.9	40.9	Palta and Gregory^43^
Wheat	151		71.4	17.7	10.9	38.2	Swinnen *et al*.^11^
Wheat	193		91.3	6.7	1.9	22.2	Swinnen *et al*.^29^
Wheat	211		66.0	22.5	11.5	33.9	Qi and Wang^47^
Wheat	50		53.7	43.0	3.2	6.9	Atwell *et al*.^28^
Barley	67		82.4	13.5	4.1	23.4	Swinnen *et al*.^29^
Barley	70		89.2	9.9	0.9	8.7	Gregory and Atwell^24^
Barley	66		69.1	8.3	22.6	73.1	Jensen^48^
Barley	56		74.3	7.8	17.8	69.4	Jensen^46^
Barley	25		62.3	15.3	22.4	59.3	Zagel^49^
		Average	74.6 ± 3.2	15.4 ± 2.0	10.0 ± 2.5	34.7 ± 6.8	
**Wheat**	**173**	**This study**	**67.8**	**9.2**	**23.0**	**71.5**	
Wheat	133	Anthesis	97.0	1.1	1.8	62.1	Keith *et al*.^25^
Wheat	106		94.2	5.7	0.1	1.8	Gregory and Atwell^24^
Wheat	179		85.3	5.3	9.4	63.9	Swinnen *et al*.^11^
Wheat	60		79.0	16.0	5.0	23.8	Martin and Kemp^44^
Wheat	226		96.5	3.3	0.2	5.9	Qi and Wang^47^
Wheat	70		84.1	14.7	1.3	8.2	Atwell *et al*.^28^
Barley	101		93.1	1.0	5.9	85.3	Jensen^48^
Barley	86		86.1	4.6	9.3	66.9	Jensen^46^
Barley	40		66.4	9.5	24.1	71.8	Zagel^49^
Barley	105		82.3	1.8	15.9	89.7	Gregory and Atwell^24^
		Average	86.4 ± 3.0	6.3 ± 1.7	7.3 ± 2.5	47.9 ± 10.8	
**Wheat**	**194**	**This study**	**58.7**	**3.9**	**37.3**	**90.5**	
Wheat	154	Grain-filling	98.9	0.4	0.7	63.6	Keith *et al*.^25^
Wheat	120		94.2	5.6	0.2	3.6	Gregory and Atwell^24^
Wheat	207		90.9	2.1	7.0	77.1	Swinnen *et al*.^11^
Wheat	255		95.4	2.5	2.1	45.5	Swinnen *et al*.^29^
Wheat	239		86.7	2.0	11.3	84.8	Qi and Wang^47^
Wheat	86		94.7	4.7	0.7	13.1	Atwell *et al*.^28^
Barley	115		97.9	1.1	1.0	47.6	Swinnen *et al*.^29^
Barley	126		97.4	2.3	0.2	8.0	Gregory and Atwell^24^
Barley	124		98.1	0.7	1.1	61.1	Jensen^48^
Barley	105		88.1	1.9	9.9	83.6	Jensen^46^
Barley	55		72.6	6.4	21.0	76.5	Zagel^49^
		Average	92.3 ± 2.3	2.7 ± 0.6	5.0 ± 2.0	51.3 ± 9.2	
**Wheat**	**208**	**This study**	**74.7**	**1.1**	**24.2**	**95.6**	
Wheat	28 DAL^b^	Whole growth period	73.5	22.0	4.4	16.8	Chowdhury *et al*.^22^
Wheat	80–90		89.0	2.3	8.4	78.1	Martens *et al*.^34^
Wheat	141		66.3	18.6	14.5	43.8	Butterly *et al*.^33^
		Average	76.5 ± 2.3	14.4 ± 2.3	9.1 ± 2.3	46.1 ± 2.3	
**Wheat**	**224**	**This study**	**69.0**	**8.7**	**22.2**	**71.8**	

^a^Data excludes that of Martin and Kemp^41^. ^b^DAL, days after ^14^C pulse labelling.

Compared with conventional non-tracing methods, C tracers (^13^C or ^14^C) labelling method can distinguish between soil-derived and root-derived C in the soil, determine the C allocation in different crop stages and completely quantify the whole seasonal C rhizodeposition^3,17^. Continuous labelling^18,19^ is particularly appropriate for the estimation of total C transferred to the soil and belowground pools. However, highly sophisticated instrumentation required for continuous isotopic ^14^C-labelling and sampling makes field studies difficult^17^, especially for long-lived crops such as winter wheat (up to eight months). As an alternative, ^13^CO_2_ or ^14^CO_2_ pulse labelling^3,8,9^ at different growth stages provides discrete information about the temporal C dynamics associated with specific crop stages^9–11^. Pulse labelling also has the advantage of being simple and applicable under field conditions and provides seasonal dynamics of assimilate partitioning. A series of ^14^C-labelling pulses applied at regular intervals during crop growth has been proven to accurately estimate cumulative belowground C inputs^3,11^, especially in calcareous soils^11^. It was commonly considered that in conventional pulse-chase labeling studies, the distribution of assimilated C was completed at the time when the losses of labeled CO_2_ by respiration could no longer be detected^11,16^. As the plant tissues and soil microorganisms prefer to utilize the labile organic substrates, the decline or disappearance of the labeled CO_2_ respiration may not necessarily indicate that the transfer of the less labile components (such as root debris and sloughed-off cells) into the soil is also completed^20^. Therefore, due to the short duration of labelling and chasing periods, most previous conventional pulse–chase labelling studies provides information only on the newly assimilated C^3,11^.

In current study, we undertook the pulse labelling at different growth stages of the whole winter wheat season and harvested the winter wheat at 5 days after ^14^C-labelling (DAL) at each growth stage and at the end of the wheat growth season^10,15,20,21^. The ^14^C distribution in the wheat biomass and soil pools at the end of the growing season represents the net contribution of photosynthates formed at various stages of wheat^15,20,21^. We aimed to quantify the temporal ^14^C distribution in the winter wheat biomass and soil pools throughout the whole wheat season. We hypothesized that the C allocated into belowground (roots and soil) by pulse labelling quantified for the whole wheat season was much lower than that C quantified by extrapolating the C amount from the young stage to the whole wheat season. To validate this hypothesis, we labelled the winter wheat with ^14^C at key growth stages, i.e., tillering, elongation, anthesis and grain filling stages, and measured the photosynthesized C allocated into shoot, roots and soil pools and lost by respiration. The results from the pot labelling were also estimated to field level in discussion Section, to evaluate the contribution of wheat production to farmland SOC changes under agricultural intensification process.

## Results

### Biomass C of shoots, roots and whole wheat plants

**Figure 1:**
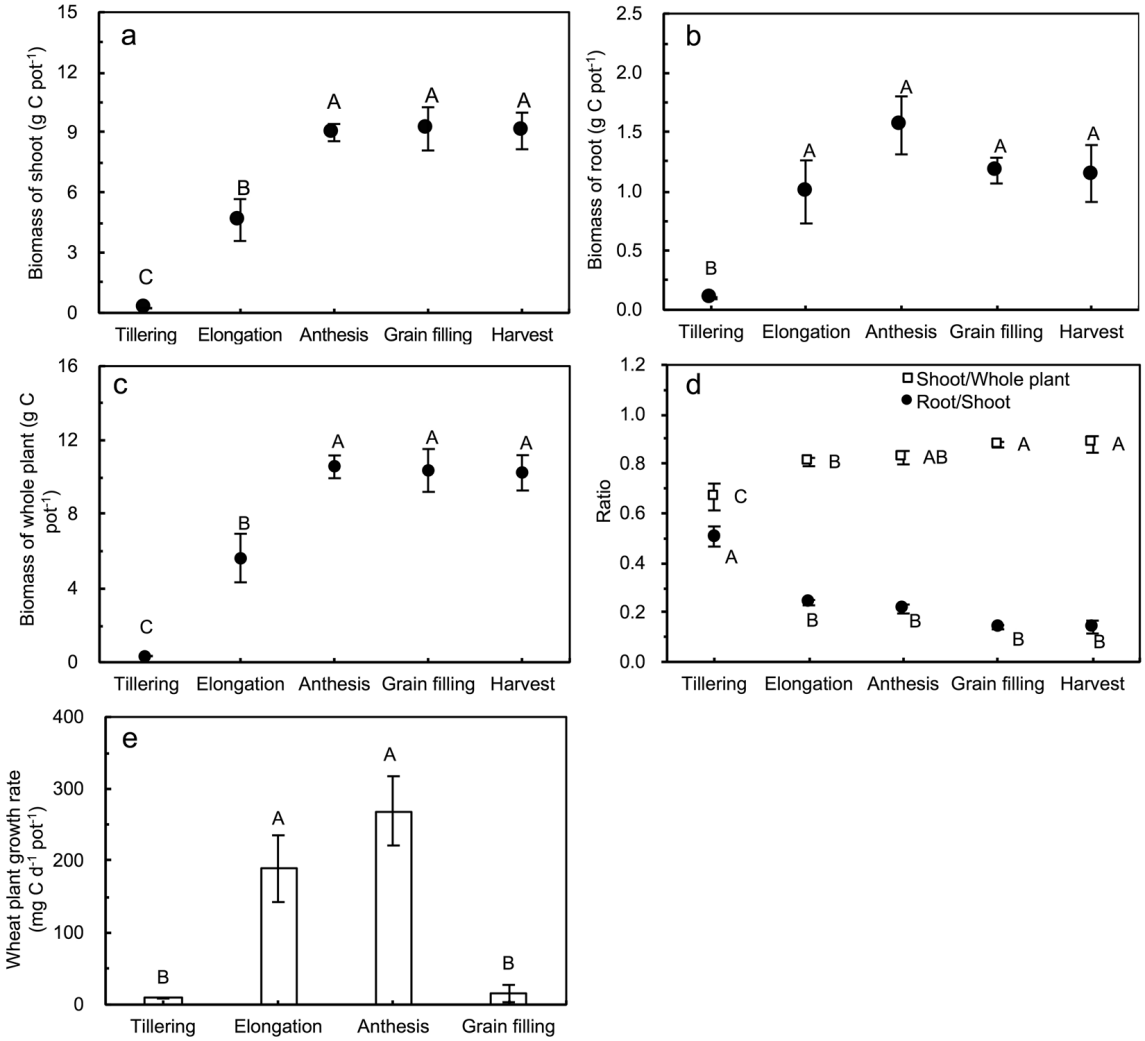
Biomass C of (**a**) shoots, (**b**) roots, (**c**) whole plants, and (**d**) the root/shoot and shoot/whole plant ratios as well as (**e**) winter wheat growth rates at different growth stages. Vertical bars represent the standard errors of the means (*n* = 3). Letters (**a**–**c**) indicate significant differences (*p* < 0.05, LSD) for comparison among different stages.

As the winter wheat plants grew, the biomass C of the shoots, roots and whole plants increased and peaked at the anthesis stage, after which it remained stable, but the biomass C of the roots decreased after the anthesis stage (Fig. 1). The ratios of roots/shoots and shoots/whole wheat plants decreased and increased, respectively, from emergence until the grain-filling stage. The growth rate of the whole wheat plants was quite low (8.8 mg C d^−1^ pot^−1^) at the tillering stage but increased to 189.0 and 269.4 mg C d^−1^ pot^−1^ at the elongation and anthesis stages, respectively; at the grain-filling stage, the growth rates were negligible.

### Specific ^14^C activity of the shoots, roots and soil

**Figure 2:**
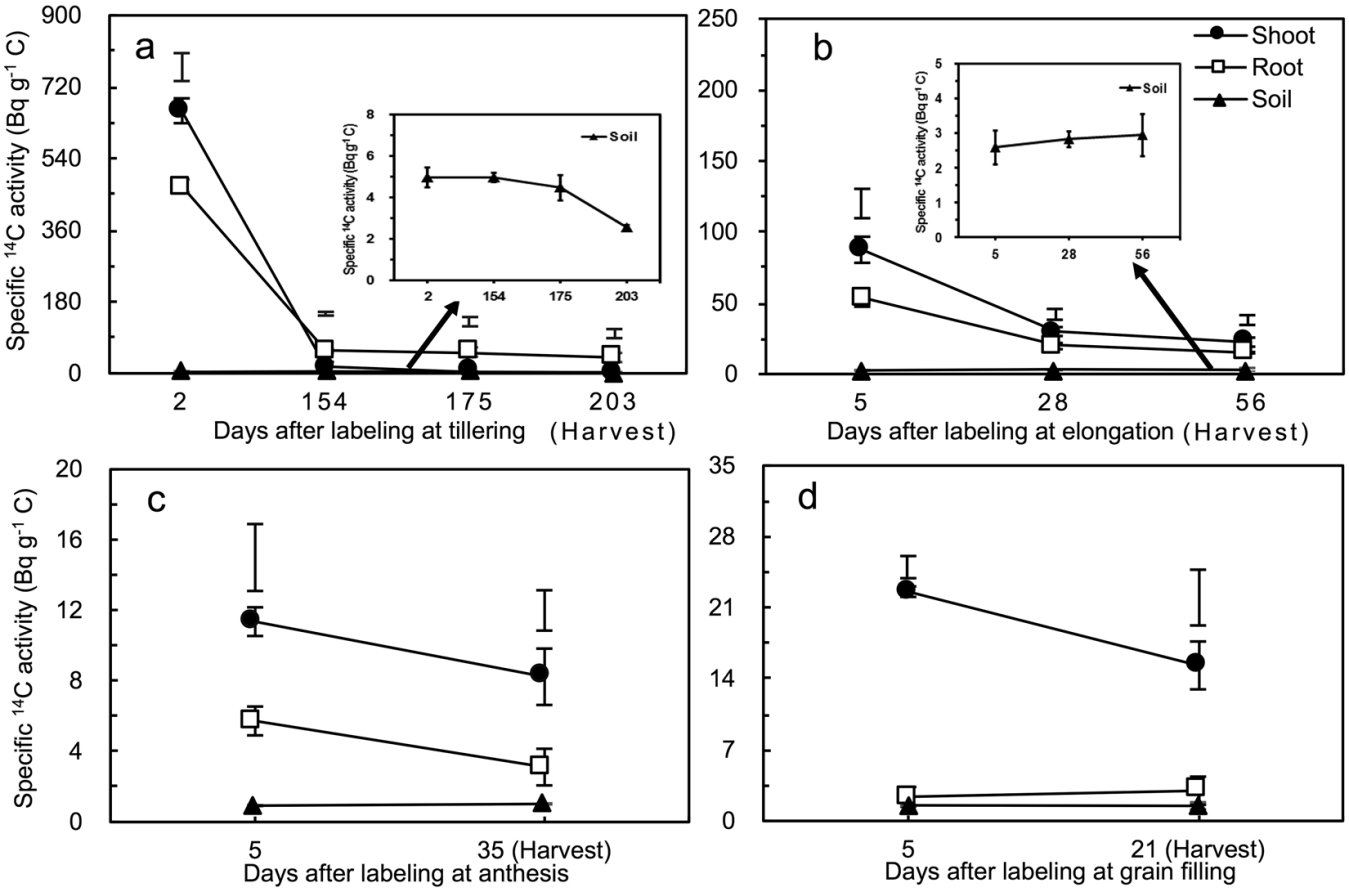
Turnover of specific ^14^C activity for shoot, root and soil components at different DAL at the (**a**) tillering, (**b**) elongation, (**c**) anthesis and (**d**) grain-filling stages of winter wheat. Vertical bars represent the standard errors of the means (*n* = 3). LSD values (*p* < 0.05) are presented as whisked segments for comparison among different components at the same stage.

Enrichment of labelled ^14^C was highest in the shoots, followed by the roots and soil for all four labelling events (Fig. 2a–d). The specific ^14^C activity of the shoots, roots and soil at 5 DAL (DAL 5; DAL 2 at the tillering stage) was in the order of tillering> elongation> anthesis ≈ grain-filling stages. With the prolonged wheat growth duration and the dilution of plant biomass and respiratory release, the specific ^14^C activities of shoots and roots decreased. For the labelling at the tillering stage, the specific ^14^C activity of the soil remained stable between DAL 2 and DAL 175 but declined thereafter (Fig. 2a); at the other three labelling stages (Fig. 2b,c,d), the specific ^14^C activity of the soil was stable after labelling.

### ^14^C recovery and allocation within the wheat–soil system

**Figure 3:**
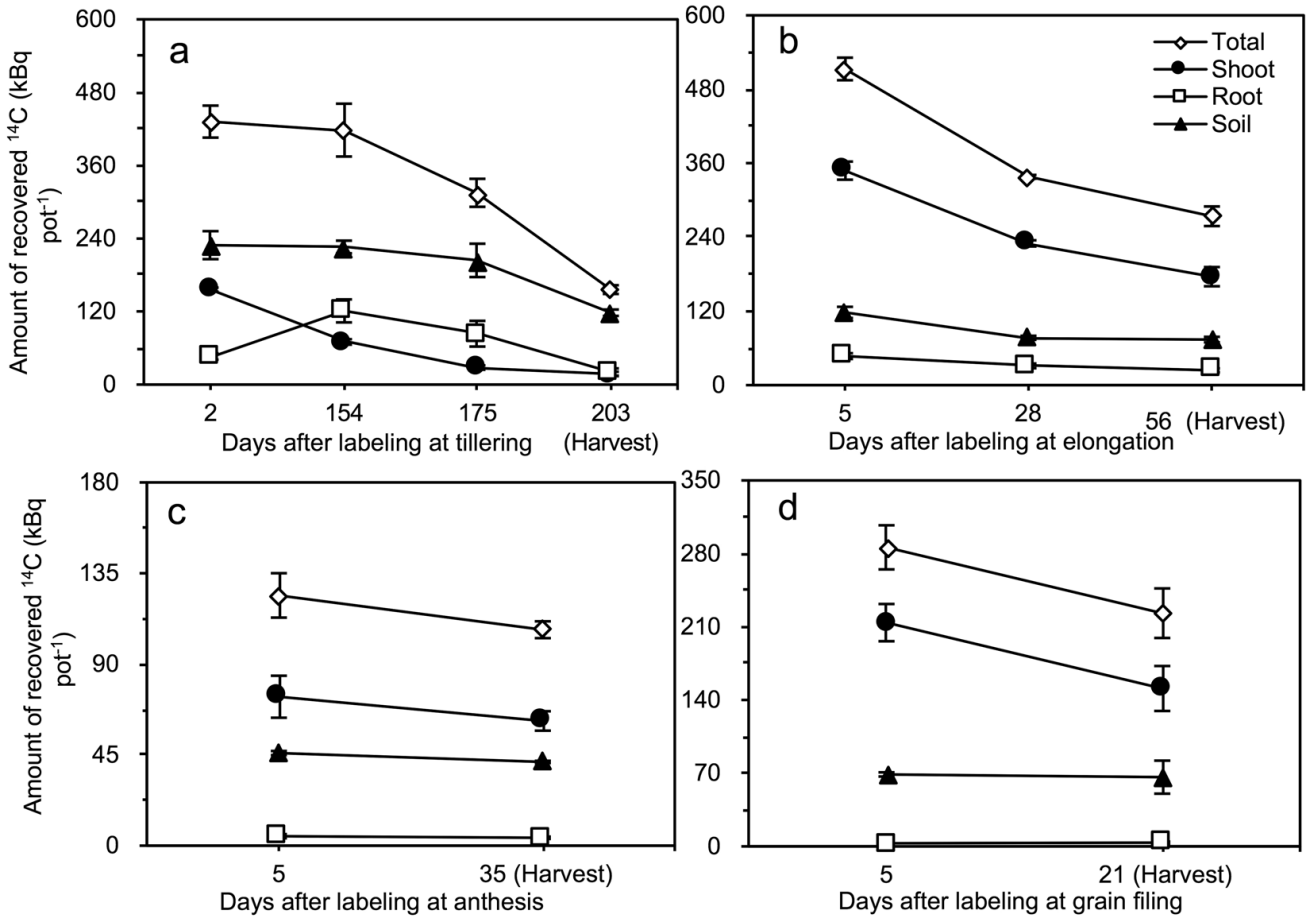
Recovered ^14^C in shoot, root and soil components at different DAL at the (**a**) tillering, (**b**) elongation, (**c**) anthesis and (**d**) grain-filling stages of winter wheat. Vertical bars represent the standard errors of the means (*n* = 3).

The ^14^C recovered in the soil was highest for the labelling at the tillering stage, followed by that in the roots and shoots (with the exception of DAL 2 in the roots; Fig. 3a). For the labelling at the other three stages, the ^14^C recovered always followed the order of shoots> roots> soil (Fig. 3b,c,d).

**Figure 4:**
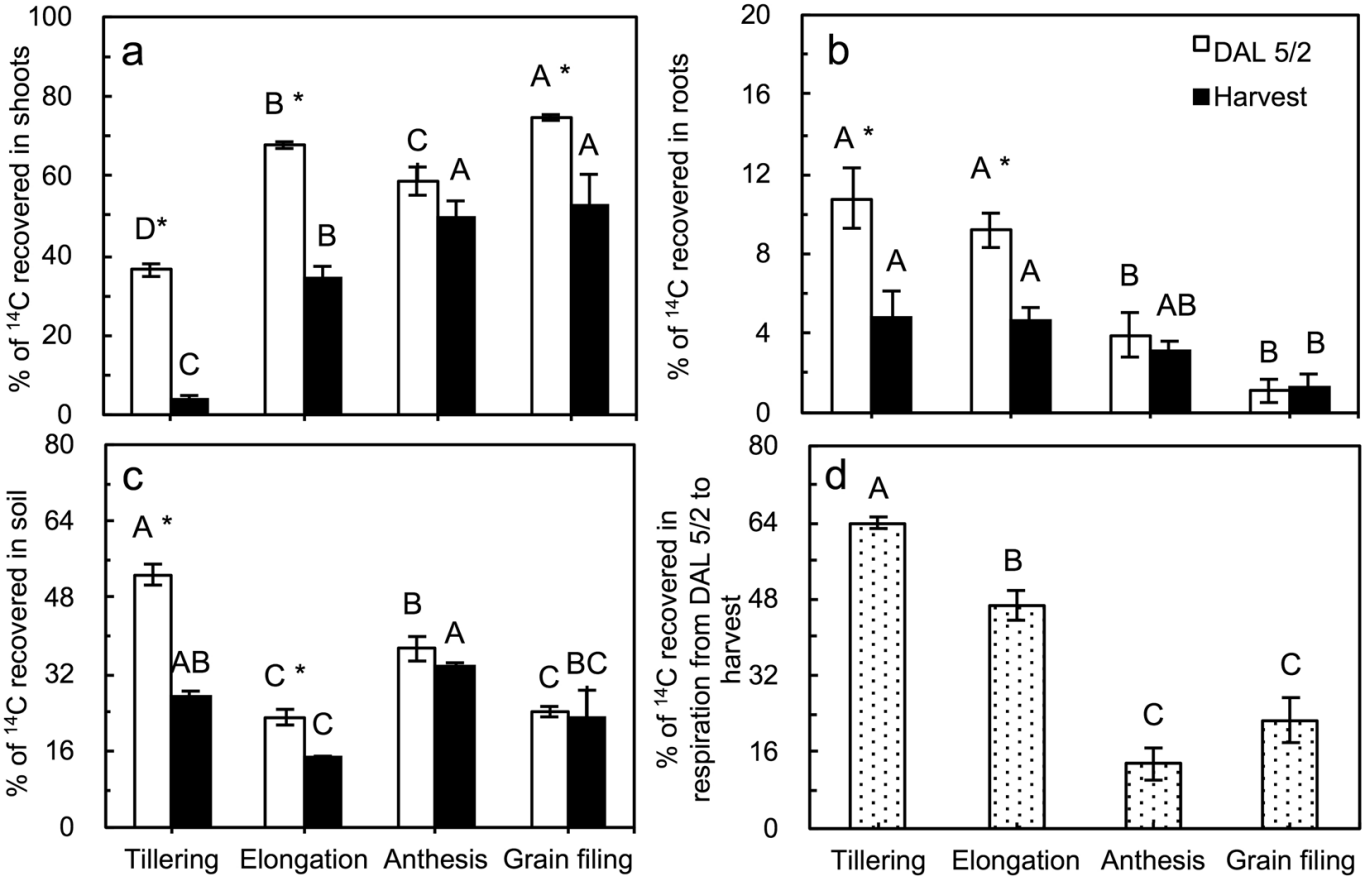
Proportion of ^14^C allocated at DAL 5/2 and at the end of the growing season (harvest) for (**a**) shoots, (**b**) roots, (**c**) soil, and (**d**) respiration at harvest for the different labelling events. Vertical bars represent the standard errors of the means (*n* = 3). Letters (**a**–**c**) indicate significant differences (*p* < 0.05, LSD) for comparison among different stages. Asterisks (*) denote significant differences (*p* < 0.05, t-test) between DAL 2/5 and harvest.

We quantified the proportion of ^14^C recovered in the shoots, roots and soil at DAL5/2 and at harvest, and that in the respired losses from DAL 5/2 to harvest. Of the total net ^14^C recovered (shoots + roots + soil), 36.4%, 67.8%, 58.8% and 74.7% were allocated in the shoots at DAL 5/2 at the tillering, elongation, anthesis and grain-filling stages, respectively; these proportions decreased to 4.0% (tillering), 34.3% (elongation), 49.9% (anthesis) and 52.9% (grain-filling) at harvest (Fig. 4a). The proportion of ^14^C in the total net ^14^C recovered in the soil (Fig. 4c) was lower than that in the shoots but higher than that in the roots (Fig. 4b; except at the tillering stage), and this proportion decreased from DAL 5/2 to harvest (Fig. 4b,c). Between DAL 5/2 and harvest, the proportion of ^14^C via respiration (aboveground and belowground respiration) was highest (63.9%) at the tillering stage but decreased to 46.7%, 13.8% and 22.1% at the elongation, anthesis and grain-filling stages, respectively (Fig. 4d). In absolute values, the amount of respired C was 0.21, 2.47, 0.51 and 0.07 C pot^−1^ at the tillering, elongation, anthesis and grain-filling stages, respectively.

### Contribution of photosynthesized C to SOC

**Figure 5:**
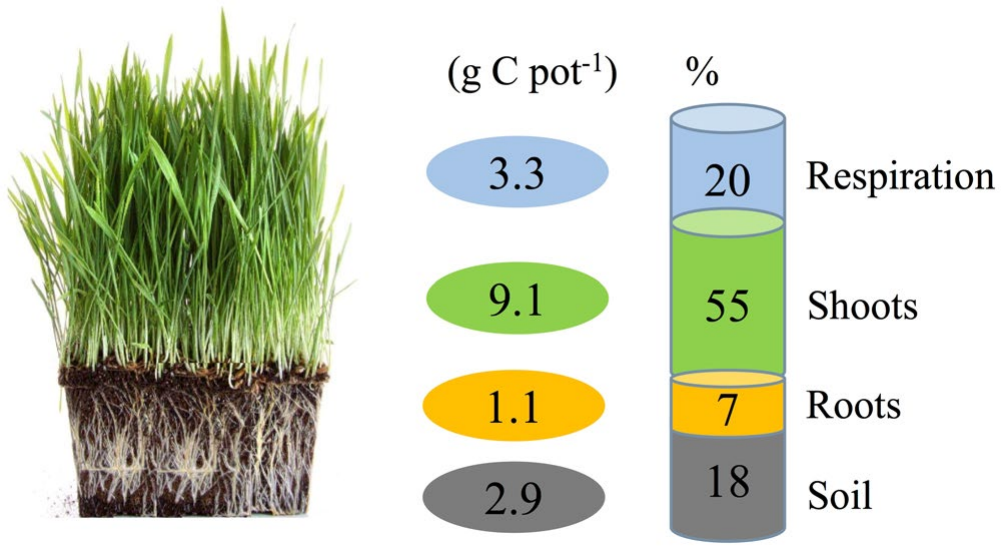
Total carbon distribution in the wheat-soil pot system.

At the end of wheat growing season, the contribution of photosynthesized C to SOC at the four growth stages was calculated by multiplying the relative distribution of assimilated ^14^C in the soil to the increment of wheat biomass C (Eq. (3)). Approximately 3.3 g C pot^−1^ was respired (i.e., 20% of the total photosynthate C recovered), 9.1 g C pot^−1^ (55%) remained in the shoots, 1.1 g C pot^−1^ (7%) was in the roots, and 2.9 g C pot^−1^ (18%) was translocated to the soil (Fig. 5). Regarding the 2.9 g C pot^−1^ allocated to the soil, contributions of 42.1% and 48.5% occurred during the elongation and anthesis stages; these percentages were significantly higher than those at the tillering (6.4%) and the grain-filling (3.0%) stages.

## Discussion

Assimilated C is translocated to plant–soil compartments such as shoots and roots, exuded into the soil and lost due to respiration, and this allocation pattern changes with crop growth^15,20–22^. In the present study, the assimilated ^14^C in the winter wheat shoots at the labelling event of tillering stage was only 36.4%, but increased at the later labelling events: i.e., the shoots retained the majority (60–74.7%) of their assimilated ^14^C at DAL 5 during the elongation, anthesis and grain-filling stages (Fig. 4). Similar results were also reported for rice^20,23^, wheat, barley^22,24,25^ and maize^16^ but not for pasture grasses^26^. Several reasons could explain the higher proportion of assimilated C allocated belowground in pasture plants than in cereal plants: (i) approximately 80% of pasture plants are perennial and have well-developed roots that are used as for C storage for newly grown plant tissues; (ii) a long history of selective cultivation of cereals has led to the preferential allocation of assimilates to aboveground parts^14^; and (iii) intensive fertilization of crops significantly decreases the assimilates in roots needed for the uptake of soil nutrients^3,27^. These findings are consistent with those of other studies (Table 1), which have reported that wheat and barley allocate more than 90% of photosynthates to their shoots during the mature stage in comparison with approximately 60% during the tillering stage. In a broader context, this indicates that different types of crop have various potentialities of atmospheric C sequestration^14^.

Photosynthates are actively translocated to belowground parts more during the early establishment of wheat and barley crops than at the mature stage^28–30^ (Table 1). The summary for previous labelling studies (Table 1) showed that the C sink in the roots was greater in young plants (27%) than in mature plants (3%) (Table 1). In our study, the percentage of ^14^C incorporated into the roots also decreased from the tillering to the grain-filling stages (Fig. 4). However, as the wheat growth rate at the anthesis and elongation stages (189−269 mg C d^−1^ pot^−1^) was much greater than that at the grain-filling and tillering stages (8.8−15.4 mg C d^−1^ pot^−1^), C translocation to the soil also occurred mainly at the anthesis and elongation stages, i.e., 48.5% and 42.1% of the total C was translocated to the soil, respectively. The majority of labelling studies have been conducted at the early growth stages of wheat, e.g., 60 days after emergence by continuous labelling^31,32^ and <150 days after emergence by pulse labelling^33,34^. Hence, extrapolating the values from early wheat stages to the whole lifetime of wheat plants (approximately 240 days after emergence in northern China) substantially overestimated the amount of C allocated to the belowground pool, as young plants exhibit faster root growth and greater sink strength of C allocated to the roots and soil than do older plants^15,16^.

Using a field planting density of 1.8 million plants ha^−1^ in northern China^35^, we found that the assimilated C input to the soil, excluding the C allocated to the roots (690 kg C ha^−1^), was approximately 1730 kg C ha^−1^ throughout the whole winter wheat season. This estimate is higher than reported 300 kg C ha^−1^ for maize^16^, 460–822 kg C ha^−1^ for rice^23^, and 710–1020 kg C ha^−1^ for wheat^36^. If the C retained as root residue after harvest (690 kg C ha^−1^) was also included in the total C input to the soil, the total C input belowground by winter wheat was 2420 kg C ha^−1^, which is significantly higher than both the values (992 kg C ha^−1^) summarized in Table 2 and the rough estimates (1500 kg C ha^−1^) by Kuzyakov and Domanski^3^ based on C tracer studies. The differences between our study and other studies might be related to the higher root biomass C resulting from agricultural intensification in our study than the quantity defined using tracer techniques (690 in our study vs. 402 kg C ha^−1^ in Table 2). In addition, the ratio of net rhizodeposited C into the soil to root was reported to be 25% by Kuzyakov and Domanski^3^ and was therefore 2.5-fold lower than that in our study. This result is because most of the estimates of Kuzyakov and Domanski^3^ for wheat labelling have been carried out at young plant stages, during which translocation is relatively higher than that during subsequent stages of growth. Again, this finding validates our hypothesis and highlights that labelling and sampling throughout the entire growing season of winter wheat (for instance> 200 days in our study) are necessary to accurately quantify the C budget within the crop–soil system.

**Table 2:** Amount of carbon (C) translocated belowground (mean ± SE) for wheat and barley quantified by C pulse labelling methods.

	Plant age (days)	Roots (kg C ha^−1^)	Soil (kg C ha^−1^)	Roots + soil (kg C ha^−1^)	References
Wheat	167	327	16	343	Gregory and Atwell^24^
Wheat	63	231	560	791	Martin and Merckx^50^
Wheat	288	350	730	1080	Swinnen *et al*.^29^
Wheat	239	840	526	1366	Qi and Wang^47^
Barley	167	324	24	348	Gregory and Atwell^24^
Barley	127	449	818	1267	Jensen^48^
Barley	148	430	880	1310	Swinnen *et al*.^29^
Barley	95	264	1171	1435	Jensen^46^
	Average	402 ± 68	591 ± 143	992 ± 158	
**Wheat**	**224**	**690**	**1730**	**2420**	**This study**

Agricultural intensification has not only increased crop productivity but also contributed to increased SOC in northern China^12^. Smith *et al*.^37^ reported that inputs of crop residues lead to higher rates of C sequestration (0.7 Mg C ha yr^−1^) than does mineral nitrogen (N) fertilizer (0.2 Mg C ha yr^−1^). The estimated wheat C inputs in our study were 2420 kg C ha^−1^ (soil + root) and 1730 kg C ha^−1^ (soil only), which were two-fold greater than those of comparable studies (Table 2). The high amount of C allocated to belowground plant parts in this calcareous soil might explain the rapid increase in SOC in northern China^12^.

## Conclusions

During a growing season, the estimated photosynthesized C input from wheat to the soil was 1.7 t C ha^−1^ as rhizodeposits. Wheat plant also produced 0.7 t C ha^−1^ of root residues that was retained in the soil after harvest. Therefore, approximately 2.4 t C ha^−1^ of atmospheric CO_2_ was fixed as a relatively stable form in the soil after the wheat season; this value is approximately twice that of other comparable studies. Of the wheat C input to the soil, 90.6% was photoassimilated between the elongation and anthesis stages. This information will be critical in the construction of predictive models of C dynamics in wheat–soil systems if the amount of plant C transferred to the soil requires estimation.

## Materials and Methods

### Experimental setup

Soil samples were collected from the ploughing layer (0–30 cm) of crop fields at the Quzhou Experimental Station of China Agricultural University in Hebei Province, China (36˚52′N; 115˚01′E). The main soil properties were as follows: 17.1 g kg^−1^ SOC, 7.8 g kg^−1^ soil inorganic C, 1.6 g kg^−1^ total N, pH 8.0 (soil/water = 1/2.5), 148 mg kg^−1^ available potassium (K), and 9.9 mg kg^−1^ Olsen phosphorus (P). The soil was sieved (5 mm), after which each polyvinylchloride (PVC) plastic pot was filled (height: 50 cm, inner diameter: 10 cm; 5.6 kg soil pot^–1^ with calculated bulk density of 1.42 g cm^−3^) and rewetted to 65% of the water-holding capacity (0.21 g water g^−1^ dry soil).

Winter wheat (*Triticum aestivum* L. cv. Jingdong 8) seeds were surface-sterilized in 30% hydrogen peroxide for 30 min, after which they were soaked for 6 h in saturated copper sulphate solution and rinsed with deionized water. This pretreatment effectively sterilized seeds and improved germination rates^38^. After disinfection, the seeds were placed into glass Petri dishes lined with wet filter paper and germinated for 2 days at 22 °C in darkness. Six healthy germinated winter wheat seeds were transplanted to an individual pot (2-cm depth below the soil). One week after wheat germination, three vigorous seedlings were kept in each pot, i.e., equivalent to a field density of 1.8 million plants per ha, which was estimated from local wheat fields. In accordance with local farming practices, the soil was premixed with urea, diammonium phosphate and potassium chloride at rates of 0.15 g N, 0.09 g P and 0.25 g K kg^−1^ soil as a base fertilizer, respectively. Urea was top-dressed at the elongation stage (mid-March of the following year) at a rate of 0.15 g N kg^−1^ soil.

The pots with transplanted winter wheat were placed inside a greenhouse to provide light and temperature conditions similar to those in the field. The soil water content of each pot was controlled gravimetrically to simulate local wheat production and was adjusted daily to 65% (during the seedling stage), 70% (tillering), 80% (elongation), 80% (anthesis) and 70–75% (grain-filling) of field water-holding capacity. When the daily average air temperature dropped to 4–5 °C during the winter season, the pots with wheat plants were placed underground (50-cm depth) to prevent cold damage. The total wheat growing period was 230 days, and six different growth stages were recognized in terms of days after sowing (DAS): (i) seeding (1–17 DAS); (ii) tillering (18–38 DAS); (iii) wintering (39–150 DAS); (iv) elongation (151–179 DAS); (v) anthesis (180–193 DAS); (vi) grain-filling (194–214 DAS); and (vii) harvest (215–230 DAS).

### ^14^C pulse labelling

^14^CO_2_ labelling was performed at the tillering, elongation, anthesis, and grain-filling stages (i.e., 21, 168, 189 and 203 DAS, respectively). In total, 33 pots of wheat were labelled: 12 pots were labelled at the tillering stage (21 DAS) for destructive sampling at days 2, 154, 175 and 203 (harvest) after labelling (3 pots per sampling); nine pots were labelled at the elongation stage (168 DAS) for destructive sampling at days 5, 28 and 56 (harvest) after labelling; six pots were labelled at the anthesis stage (189 DAS) for destructive sampling at days 5 and 35 (harvest) after labelling; and six pots were labelled at the grain-filling stage (203 DAS) for destructive sampling at days 5 and 21 (harvest) after labelling.

The labelling system adapted from Cheng^39^ consisted of a labelling chamber and a pot containing transplanted wheat. Each pot was closed at the bottom with a rubber stopper and had an air inlet and air outlet. The soil surface was covered with a PVC board and sealed with silicon, including around the winter wheat stems. A flask containing Ba^14^CO_3_ with a ^14^C activity of 713 kBq was placed inside the labelling chamber. The chamber was then closed, and ^14^CO_2_ was released into the chamber by carefully adding an excess volume of 4 *M* HClO_4_ to the Ba^14^CO_3_ solution with a syringe to ensure complete evolution of the ^14^CO_2_ into the labelling chamber atmosphere. To help guarantee a uniform distribution of ^14^CO_2_, an electric fan was used to homogenize the gases inside the chamber.

CO_2_ depletion under identical conditions in another chamber supplied with unlabelled CO2 was monitored using an infrared gas analyser (GXH305, Beijing Analytical Equipment Co., China)^40,41^. This system was used because ^14^CO_2_ could not be directly monitored, as the infrared range was set for ^12^CO_2_ and only slightly overlapped with the range for ^14^CO_2_^40,41^. If the speed of CO_2_ concentration decrease slowed considerably (less than 200 μL L^−1^), ^12^CO_2_ was released into the chamber until the ^14^CO_2_ + ^12^CO_2_ concentrations increased to approximately 400 μL L^−1^ by adding with a syringe an aliquot of 4 *M* HClO_4_ to an unlabelled NaHCO_3_ solution. The wheat plants were labelled for 1.5 h to facilitate the assimilation of ^14^CO_2_. After Ba^14^CO_3_ labelling, the air within the labelling chamber was pumped for 0.5 h through 50 mL of 1 *M* NaOH solution to remove unassimilated ^14^CO_2_ before the labelling chamber was opened.

### Plant and soil sampling

Destructive sampling of wheat and soil samples was carried out for the C and ^14^C analyses. The shoots were cut at the base of the wheat plants, and all the soil was removed from the pots. The roots were separated from the soil manually and then washed with 125 mL of deionized water to remove the soil adhering to the roots^42^. The soil samples were placed onto thick paper (0.5 cm) and divided into 100 groups. Approximately 1 g per group was sampled with a spoon and mixed to obtain a representative soil sample^38^. The shoots, roots and soil samples were oven-dried at 65 °C to a constant weight.

### Sample analysis

The plant and soil samples were ground (<500 µm) using a ball mill (Restol MM2000, Retsch, Haan, Germany) prior to analysing ^14^C content and determining total organic C. To determine the ^14^C content of SOC, carbonates were removed from the soil samples using a 0.5 *M* HCl solution for 6 h^4^. The soil was then washed using deionized water and centrifuged three to four times to remove the HCl. The ^14^C content of the plant (approximately 0.2 g) and soil (approximately 1 g) samples was measured after combustion with an oxidizer unit. The evolved ^14^CO_2_ was directly trapped in a scintillation cocktail, followed by liquid scintillation counting (FJ-2101, Xi’an Analytical Equipment Co., Xi’an, China). The ^14^C counting efficiency was approximately 94%.

### Calculations and statistical analysis

At 2 DAL (DAL 2) at the tillering stage or 5 DAL (DAL 5) at the elongation, anthesis and grain-filling stages, we assumed that the allocation of ^14^C photosynthesized within the wheat–soil system represented the initial photosynthate distribution in the shoot, root and soil compartments^15,20,21^. At each sampling, the amount of net ^14^C recovered (Fig. 3) was calculated by multiplying the specific ^14^C activity (Fig. 2) by the corresponding C amount in the shoots, roots or soil (Fig. 1). The net ^14^C recovered at DAL 5 or 2 (DAL 5/2) served as the basis for the calculation and comparison of photosynthesized C distributed in different wheat–soil compartments during the later days of wheat growth and at the harvest period.

The (Net ^14^C recovered)_DAL5/2_ of winter wheat was estimated by summing the ^14^C amount in the shoots (^14^C_shoot_), roots (^14^C_root_) and soil (^14^C_soil_) measured at DAL 5/2 at each stage^16–18^:

1 $${({\rm{Net}}{}^{14}{\rm{C}}{\rm{recovered}})}_{\mathrm{DAL}5/2}={({}^{14}{\rm{C}}_{\mathrm{shoot}}+{}^{14}{\rm{C}}_{\mathrm{root}}+{}^{14}{\rm{C}}_{{\rm{soil}}})}_{\mathrm{DAL}5/2}$$

The per cent distribution of ^14^C recovered (Distribution%) either at DAL 5/2 or at the end of the wheat growing season was calculated as:

2 $$\mathrm{Distribution} \% =\frac{{}^{14}{\rm{C}}_{\mathrm{sample}\,}}{{({\rm{Net}}{}^{{\rm{14}}}{\rm{C}}{\rm{recovered}})}_{\mathrm{DAL}5/2}}$$

where ^14^C_sample_ is the ^14^C amount in the measured pools, i.e., the shoots, roots, and soil and the respired CO_2_ (aboveground and belowground) at DAL 5/2 or at the end of the growing season.

The whole wheat seasonal contribution (g C pot^−1^ soil) of photosynthesized C to the soil formed during the four growth stages of winter wheat was calculated by the relative distribution of ^14^C recovered in the soil to the accumulation of plant biomass C as follows:

3 $${\rm{Contribution}}\,({\rm{C}}\,{{\rm{pot}}}^{-1}\,\mathrm{soil})=\frac{{({}^{14}{\rm{C}}_{soil})}_{{\rm{end}}}}{{({\rm{Net}}{}^{14}{\rm{C}}{\rm{recovered}})}_{{\rm{DAL}}5/2}}\times {\rm{WPGR}}\times {\rm{D}}$$

where (^14^C_soil_)_end_ is the amount of ^14^C in the soil at the end of wheat growing season; (net ^14^C recovered)_DAL 5/2_ is calculated by Eq. (1); WPGR is the wheat plant growth rate (mg C d^−1^ pot^−1^; Fig. 1e); and D represents the number of days in each growth stage. Seventeen days of the seeding stage was considered to encompass tillering, i.e., a total of 38 days at the tillering stage. Within 111 days of the wintering period, wheat stopped growing, and its growth rate was considered 0 mg C d^−1^ pot^−1^. The duration of the elongation, anthesis and grain-filling stages was 29, 14 and 21 days, respectively.

The respired C assimilation (from the shoots, roots and soil) was estimated from the accumulation of plant biomass C and from the ^14^C distribution by respiratory losses at the end of the wheat growing season. The amount of respired C (aboveground and belowground) was calculated as follows:

4 $${\rm{Respiratory}}\,{\rm{C}}\,({\rm{C}}\,{{\rm{pot}}}^{-1})=\frac{{({}^{14}{\rm{C}}_{{\rm{total}}{\rm{respiration}}})}_{{\rm{end}}}}{{({\rm{Net}}{}^{14}{\rm{C}}{\rm{recovered}})}_{\mathrm{DAL}5/2}}\times {\rm{WPGR}}\times {\rm{D}}$$

where (^14^C_total respiration_)_end_ indicates ^14^C-CO_2_ losses from total respiration calculated by the difference between the net ^14^C recovered at DAL 5/2 and the amount of ^14^C remaining in the wheat–soil system at the end of the growing season.

### Statistical analysis

Winter wheat growth stage was the experimental factor in this study. The experiment was carried out involving three replicates and was arranged in a completely randomized design. The data were subjected to one-way analysis of variance (ANOVA) using SPSS (Version 11.0, 2002, SPSS Inc., USA). Fisher’s least significant difference (LSD; *p* < 0.05) was used to test differences in the measured variables among the different labelling events.
